# Has the COVID-19 Pandemic Worsened Health-Related Quality of Life of Patients with Inflammatory Bowel Disease? A Longitudinal Disease Activity-Controlled Study

**DOI:** 10.3390/ijerph20021103

**Published:** 2023-01-08

**Authors:** Ilenia Rosa, Chiara Conti, Luigia Zito, Konstantinos Efthymakis, Matteo Neri, Piero Porcelli

**Affiliations:** 1 Department of Dynamic and Clinical Psychology, and Health Studies, “Sapienza” University of Rome, 00185 Rome, Italy; 2 Department of Psychological, Health, and Territorial Sciences, University “G. d’Annunzio” of Chieti-Pescara, 66100 Chieti, Italy; 3 Department of Medicine and Aging Sciences, University “G. d’Annunzio” of Chieti-Pescara, 66100 Chieti, Italy

**Keywords:** COVID-19, disease activity, health-related quality of life, inflammatory bowel disease

## Abstract

The present longitudinal study aimed to investigate the burden of disease activity change on health-related quality of life (HRQoL) of patients with inflammatory bowel disease (IBD) during the two different pandemic waves in 2020 and 2021. A sample of 221 IBD patients (recruited during March–May 2020 for T0 and March–May 2021 for T1) was included. The psychological impact of the COVID-19 pandemic (Impact of Event Scale-Revised (IES-R)) and HRQoL (Inflammatory Bowel Disease Questionnaire (IBDQ)) were assessed. Post-traumatic COVID-19-related symptoms (IES-R) were not significantly different across the disease activity-related groups. Conversely, IBDQ was consistently higher in patients with persistent, quiescent disease activity compared to the other groups, as expected. Even after controlling for baseline IES-R, repeated-measures ANCOVA showed a non-significant main effect of time (*p* = 0.60) but a significant time-per-group interaction effect with a moderate effect size (η^2^ = 0.08). During the two different phases of pandemic restrictions, IBD-specific HRQoL was modified by disease-related factors such as disease activity, rather than by the post-traumatic symptoms of COVID-19. This lends further weight to the need for developing an evidence-based, integrated, biopsychosocial model of care for patients with IBD to identify subjective and objective factors that affect the burden of disease.

## 1. Introduction

The severe acute respiratory syndrome coronavirus-2 (SARS-CoV-2) pandemic represents the most significant epidemiological event in modern human history. Among the several problems determined by the recent COVID-19 pandemic, the redefined delivery of medical care for non-critical and non-urgent procedures might have added psychological distress in patients with chronic diseases, including inflammatory bowel disease (IBD), who need regular assessments at hospitals [1]. IBD is a chronic intestinal disease of unknown etiology (most likely a combination of genetic predisposition and an abnormal immune response) whose main clinical forms are ulcerative colitis (UC) and Crohn’s disease (CD) [2]. It is characterized by intermittent phases of acute relapses and periods of quiescence. The most common acute symptoms are intestinal, such as abdominal pain, blood in the stools, and diarrhea, but patients may suffer also from extraintestinal symptoms, such as skin, ocular, and joint disorders. IBD is a chronic disabling condition that negatively impacts the physical, psychological, familial, and social dimensions of affected patients [3].

Health-related quality of life (HRQoL) is a complex, multidimensional construct that includes physical, psychological, and social domains, as well as an individual’s perceptions of their overall well-being. Chronic disease, injury, treatment, and health policy can influence HRQoL [4]. 

The association between IBD, psychological symptoms, and impaired HRQoL is well-established [5,6,7,8,9,10,11]. Due to the bidirectional brain–gut axis, psychological distress and disease activity (DA) were seen to be consistently and bidirectionally linked over time [12,13,14] and, in consequence, have a profound impact on IBD patients’ HRQoL [15,16]. Although the disease characteristics, especially DA, are important determinants of HRQoL in IBD [15,17,18,19], even quiescent patients report an impaired HRQoL, suggesting a role for other determinants [20,21,22]. The presence of psychological distress represents an important risk factor for the deterioration of HRQoL and for IBD-related negative outcomes [7]. The stress evokes hyperactivity of the midcingulate cortex, and this could intensify the symptoms of the disease and the autonomic and neuroendocrine regulation of inflammation producing an increase in inflammatory activity [23,24,25]. Neuroimaging studies have identified greater neural activity in patients with CD, supporting the hypothesis that stress evokes functional alterations in the neural structures of the brain–gut axis in Crohn’s patients [23]. The impairment of social interactions is an additional aspect that contributes to low HRQoL [26] and seems to be bi-directionally associated with the important prevalence of distress in IBD. High levels of psychological distress inhibit mentalization skills and this compromises understanding one’s own feelings and those of close others and activation of the attachment system [27]. Due to the difficulties related to disease, the activation of attachment behaviors may become dysfunctional in the context of low mentalization. IBD patients who present with an avoidant attachment, to suppress anxiety and worries that are disease-related, may withdraw from interpersonal relationships and seek excessive isolation [28]. Conversely, patients with an anxious attachment would present with insistent requests for help or demonstrations of suffering. In all cases, IBD-related stress and low mentalization negatively affect daily social interactions, patients’ close interpersonal relationships, and HRQoL [28,29].

In IBD research, it is often impossible to complete assessments required to calculate DA scores and/or assess for endoscopic remission. Therefore, both research studies and drug authorities such as the Food and Drug Administration, support the use of Patient-Reported Outcome (PRO) measures [30,31]. PROs are direct information from patients about their health condition and therapy without interpretation by health care professionals or anyone else. PRO measures can assess symptoms, signs, functional status, and perceptions [32]. During the COVID-19 emergency, there were complications associated with clinical data collection for the assessment of the health status of IBD patients, and therefore the use of PROs was increased. Recently, some studies have used these measures for investigating the effects of COVID-19 on the clinical status of patients with physical problems [33] and IBD [34]. 

In the last year, peer-reviewed journals published many editorial and commentary articles expressing concern about the potential impact of the COVID-19 pandemic on the safety and management of patients with IBD during the pandemic [35,36,37,38]. Results from empirical studies are, however, controversial. Some investigations found that, during the stay-at-home period, up to one third of IBD patients reported poor HRQoL and moderate to severe anxiety and depressive symptoms [39,40,41,42,43] whereas, in contrast, other investigations showed that disability and HRQoL were unaffected by the COVID-19 pandemic [44,45,46]. The conclusions of those previous studies are, however, limited by the lack of a control for DA. In fact, our preliminary cross-sectional study, conducted 2–3 months after the WHO pandemic declaration, found that poorer IBD-related HRQoL during the first pandemic wave (March–May 2020) was independently and more strongly predicted by the combination of somatic symptoms of IBD relapse and psychological distress rather than the pandemic-related living conditions [47]. Furthermore, to our knowledge, no study has longitudinally examined the burden of concomitant IBD activity on HRQoL during the two different waves (2020/2021) of the COVID-19 pandemic. 

The present longitudinal study aimed to investigate the burden of DA change on patients’ HRQoL by addressing some previous limitations. First, we evaluated HRQoL in two different time windows corresponding to the first (March–May 2020) and the second wave (March–May 2021) of the pandemic period. Second, the change of DA over time was used as control for the psychological status of patients. Although no previous data are available, we might expect that HRQoL would be more associated with DA than the distress related to the pandemic period. 

## 2. Materials and Methods

### 2.1. Participants and Procedure

Participants were recruited from a convenience sample comprised of those who participated in the first assessment period during the first pandemic period (T0, March–May 2020). Details of the first recruitment procedure are available elsewhere [47]. Patients enrolled in this pandemic period were contacted through emails and invited to participate in a second assessment period (T1, March–May 2021). A second email invitation was sent if no feedback was received after one month. The second assessment procedure was the same as the first assessment period. Briefly, patients were invited to compile an online survey on the Qualtrics platform (www.qualtrics.com/it, accessed on 15 April 2020) through the dissemination of advertisements on several Italian IBD patients’ groups on social media.

Inclusion criteria were the following: having been diagnosed with UC or CD by a certified gastroenterologist specialist, aged 18–75, currently speaking Italian, being in a regular medical follow-up routine at a public or private Italian hospital-based gastrointestinal care unit, received a specialist visit from the treating gastroenterologist within the last 6 months, and DA index score was available in medical record. To maximize ecological validity, exclusion criteria were surgery in the past 18 months, having a definite ileostomy, severe comorbidity (e.g., cancer, ischemic heart disease, metabolic disease, or autoimmune disease), current major psychiatric disorders (e.g., major depression, bipolar disorder, disorders in the psychotic range, etc.), mental retardation, active use of intravenous drugs, and abuse of alcohol (all based on reported medical diagnoses and pharmacological therapy), being currently positive for SARS-CoV-2 infection/COVID-19 disease, or having suffered from proven COVID-19 in the past months. 

**Figure 1:**
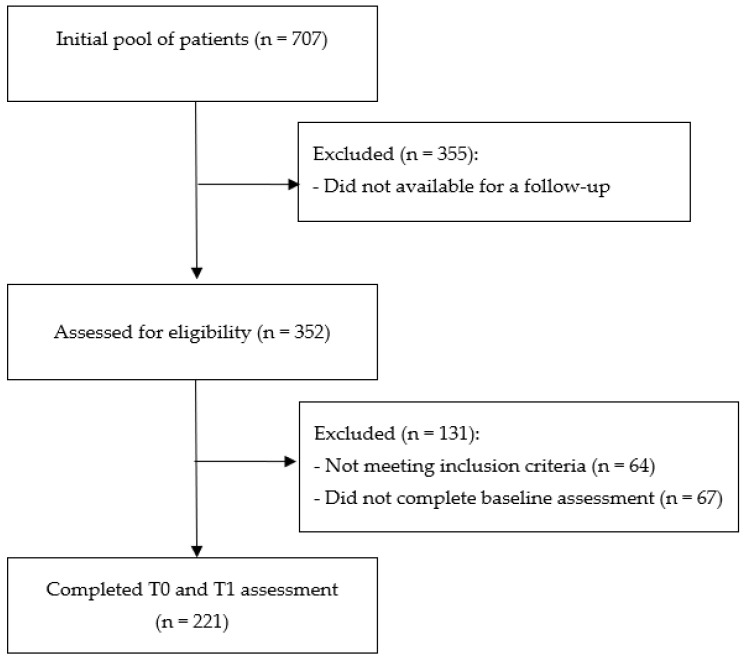
Consort diagram describing the flow of participation in the study.

From an initial pool of 707 patients [47], 352 declared their availability for a follow-up interview. Of whom, after removing those who did not satisfy the inclusion criteria and provided incomplete questionnaires, 221 (62.8%) completed two-wave data and were enrolled in the final sample of the current study (Figure 1).

All participants provided online informed consent to participate in the study and their anonymity was guaranteed. 

### 2.2. Ethical Statement

The study was designed and carried out in accordance with the World Medical Association’s Declaration of Helsinki and its subsequent revisions [48] and approved by the Ethics Committee of the Department of Psychological, Health and Territorial Sciences of University d’Annunzio, Chieti-Pescara, Italy (Protocol Number: 2020/20003).

### 2.3. Measures

#### 2.3.1. Sociodemographic and IBD-Related Clinical Variables

Sociodemographic and IBD-related clinical variables were self-reported by participants, including age, gender, educational level, and disease characteristics. Disease characteristics included the type of disease (UC or CD), the average number of annual relapses, disease duration, and DA (active or quiescent) as reported in their medical record at the most recent medical visit. The full Mayo index was used to measure DA in UC patients [49]. The Mayo score is composed of four clinical parameters: stool frequency (0 = within individual normal frequency to 3 = at least five times more than normal), rectal bleeding (0 = none to 3 = only blood evacuations), endoscopic findings (0 = normal rectal mucosa to 3 = severe bleeding), and physician’s global assessment (0 = normal health status to 3 = severe poor global health and functional impairment). The total score on the scale ranges from 0 to 12 and can be classified into four DA groups: 0–2 remission, 3–5 mild activity, 6–10 moderate activity, and 11–12 severe activity [50]. The Crohn’s Disease Activity Index (CDAI) was used to measure DA in CD [51]. The CDAI is characterized by eight independent variables: number of liquid or very soft stools, abdominal pain, general well-being, extraintestinal complications, antidiarrheal drugs, abdominal mass, hematocrit, and deviation from standard body weight [51]. CDAI values <150 indicate quiescent disease; >450 indicates extremely severe disease [52].

#### 2.3.2. Post-Traumatic Symptoms

The psychological impact of the COVID-19 pandemic was measured with the Impact of Event Scale-Revised (IES-R) Italian version [53]. IES-R is a self-report questionnaire that assesses current subjective distress in response to a specific traumatic event. It is composed of 22 items on a scale from 0 (“not at all”) to 4 (“extremely”) [54]. The IES-R has three subscales, which include intrusion (IES-IT), avoidance (IES-A), and hyperarousal (IES-H) [55], that represent three dimensions of PTSD. The total score could range between 0–88 and a score of 33 or above indicates possible PTSD [55]. The IES-R original version demonstrated good psychometric properties, with high internal consistency for the total scale (α = 0.96) [55]. During the pandemic, the IES-R was used to evaluate post-traumatic COVID-19-related symptoms (e.g., [56]). Within this sample, Cronbach’s α was 0.92 for the IES-R total score at both T0 and T1.

#### 2.3.3. Health-Related Quality of Life

The HRQoL was measured with the Inflammatory Bowel Disease Questionnaire (IBDQ) [57]. The IBDQ is a widely used self-report, disease-specific 32-item questionnaire. It is answered on a scale from 1 (indicating the highest symptom frequency/severity) to 7 (indicating the lowest symptom frequency/severity). The IBDQ measures four aspects of HRQoL in IBD patients: bowel symptoms, systemic symptoms, emotional function, and social function. The bowel symptoms subscale assesses the bowel disorder (e.g., loose stools, abdominal pain); the systemic symptoms subscale includes fatigue and sleep disturbances; the social function subscale measures presence at work and the need to avoid social events; and the emotional subscale assesses anger, depression, and irritability. The total score could range between 32–224, with higher scores representing a better quality of life [57]. In our sample, the total score range between 51–211, with low levels of HRQoL (≤25th; centile = 51–107), middle-low HRQoL (25th–50th; centile = 108–127), middle-high HRQoL (50th–75th; centile = 128–155), and high HRQoL (≥75th; centile = 156–211).

Within this sample, Cronbach’s α was 0.95 for the IBDQ total score, 0.86 for bowel symptoms, 0.83 for systemic symptoms, 0.86 for emotional function, and 0.82 for social function at T0. Cronbach’s α was 0.96 for the IBDQ total score, 0.88 for bowel symptoms, 0.83 for systemic symptoms, 0.87 for emotional function, and 0.87 for social function at T1.

### 2.4. Statistical Analyses

Between-group differences were examined using the chi-square test (χ^2^) for categorical variables (gender, educational level, disease, annual relapses, disease duration) and analysis of variance (ANOVA) for continuous variables (e.g., age, clinical characteristics). Effect sizes for categorial variables were assessed by using Cramér’s V ranging from 0 to +1, where 0 indicates a complete independence of two variables and +1 indicates a 100% perfect association. Effect sizes for continuous variables were assessed with the eta-square (η^2^). Eta-square of 0.01–0.05 is considered small, 0.06–0.14 is moderate, and >0.14 is large [58]. 

The change of HRQoL between T0 and T1 in DA groups was investigated through the repeated-measures analysis of covariance (ANCOVA). The repeated-measures ANCOVA included measures of HRQoL as a dependent variable, the time points T0 and T1 as a within-subject factor, COVID-19-related post-traumatic symptoms as covariate, and DA groups in the between-subject comparison. The level of significance was set at 95%. Statistical Package for Social Science (SPSS) version 26.0 (IBM SPSS Statistics for Windows, Version 26.0.; IBM Corp, Armonk, NY, USA) was used for all statistical analyses. 

## 3. Results

### 3.1. Characteristics of the Sample

Four DA change-related groups from T0 to T1 were formed: A-Improved (from active to quiescent), B-Worsened (from quiescent to active), C-Persistent Active (both active), and D-Persistent Quiescent (both quiescent) (see Table 1). 

Table 2 reports sociodemographic and disease-related characteristics of the total sample and the four DA change-related groups. No between-group difference was present, except the number of annual relapses (η^2^ = 0.36). As expected, patients with persistently active disease (group C) had a more severe disease course (i.e., >6 relapses per year: 23.6%, standardized deviate = +3.40; 0–1 relapse per year: 18.2%, standardized deviate = −2.54). Conversely, patients with persistent quiescent disease (group D) had a less severe disease course (i.e., 0–1 relapse per year: 74.2%, standardized deviate = +4.29; 2–5 relapses per year: 22.6%, standardized deviate = −3.11). 

Table 3 reports the clinical characteristics of the total sample and the four DA change-related groups. Post-traumatic COVID-19-related symptoms (IES-R) were not significantly different across the DA change-related groups. Conversely, IBD-specific HRQoL (IBDQ total and subscale scores) showed significant between-group differences at both assessment times, particularly at T1 when effect sizes were found in the large range (η^2^ > 0.14). Overall, post-hoc Bonferroni-corrected between-group comparisons showed that IBD-related HRQoL was associated with the disease course, as would be expected. In particular, IBDQ was consistently higher in patients with persistent quiescent disease (group D) compared to the other groups, as expected. Of interest, but also as expected, emotional symptoms were significantly higher (lower IBDQ Emotional Functions subscale score) in patients with persistent active disease.

### 3.2. Disease Activity Change-Related Groups Comparisons across Time

At the baseline, patients may have different individual levels of psychological distress that are related to non-assessed factors such as stressful events, psychosocial functioning, contextual life problems, and past experiences with illness. Since direct group comparisons (Table 3) may underestimate real inter-individual differences, the repeated-measures ANCOVA was used to compare IBDQ total score at T0 and T1 between the four DA groups while controlling for the post-traumatic COVID-19-related symptoms (Table 4). Even after controlling for baseline IES-R, the repeated-measures ANCOVA showed a non-significant main effect of time (*p* = 0.60) but a significant time-per-group interaction effect with moderate effect size (η^2^ = 0.08). Post-hoc analyses revealed a significant between-group difference in patients with persistent quiescent disease (group D) who reported higher HRQoL than the other three groups (data not shown). 

**Figure 2:**
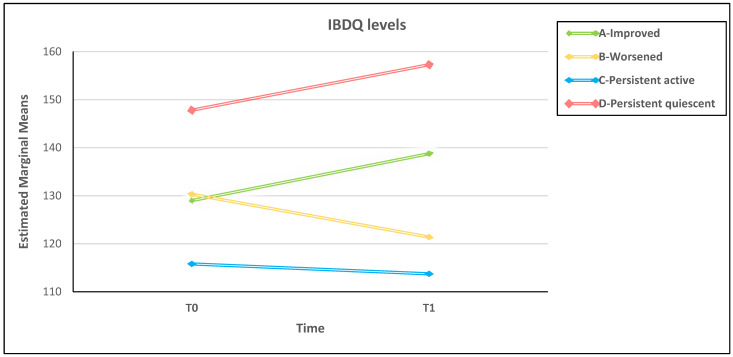
Over time HRQoL in the four disease activity change-related groups adjusted for baseline levels of post-traumatic distress.

The overall picture can be shown graphically in a clearer manner (Figure 2). The extreme levels of better and worse HRQoL were obtained by patients with unchanged DA over time (groups A–D). Conversely, in patients with changed DA, regardless of their baseline DA level, HRQoL temporal slope followed consistently the upward and downward direction of the other two extreme HRQoL-related groups (Figure 2). 

## 4. Discussion

Current research on HRQoL suggests that the construct of quality of life is not stable and invariant but is subject to several changes over time between and within individuals [10,11]. This sustains the need for longitudinal designs to identify factors that affect the HRQoL in patients affected by IBD and other chronic illnesses with unforeseeable, remitting/relapsing course. Our longitudinal study confirmed the lack of effect of COVID-19-related psychological distress on HRQoL of IBD patients. Conversely, HRQoL was strictly associated with the disease course, as expected. The unfortunate event of the COVID-19 pandemic has given the opportunity to obtain an ecological evaluation of extra-disease and disease-related factors that matter in the subjective perception of the IBD patients’ health status. Our results showed that during the two different phases of pandemic restrictions, IBD-specific HRQoL was modified by disease-related factors such as DA, rather than by the post-traumatic symptoms of COVID-19. These findings confirm the results of our preliminary cross-sectional study [47] in which COVID-19-related psychological distress only played an unexpectedly negligible role on IBD-specific HRQoL and are consistent with the research conducted during the current pandemic [44,45,46,59]. To our knowledge, the only longitudinal study conducted during the COVID-19 pandemic reported that IBD-related HRQoL remained mostly stable during different phases of restrictions [46].

Overall, the studies on HRQoL conducted during the COVID-19 pandemic in the IBD population did not consider the DA role, which may partially explain the inconsistent results in literature on the psychological consequences of COVID-19 restrictions in IBD patients. In our study, better and worse HRQoL levels were obtained by patients with persistent quiescent and active disease, respectively, whose DA did not change from T0 to T1. Between IBD patients with persistent quiescent and active disease (see Figure 2), there were patients with DA that changed over time who entered the study with a similar HRQoL level. Regardless of their DA at baseline, these patients reported different paths of HRQoL over time. Although the HRQoL never reached extreme levels, improved patients reported an increase in HRQoL over time and worsened patients reported a decrease. The unstable disease course of improved and worsened IBD probably leads patients to deal with the uncertainty of the symptoms and the unpredictability of the illness. It is known that the sense of uncertainty and uncontrollability of a disease condition can impair HRQoL [60,61,62,63]. One may speculate that uncertainty causes stress [64,65] that, in turn, may contribute to acute symptoms of IBD [66] and further impairment of HRQoL. In summary, the changing HRQoL is a result of disease course and illness instability [67,68,69,70,71].

The major strength of this study was the longitudinal design that allowed us to assess the progression of HRQoL throughout the pandemic. However, there are also some limitations to acknowledge. First, the pre-lockdown level of HRQoL of participants was unknown. This did not allow us to measure the actual size of the impact, an issue that comes to light in some of the other studies mentioned herein [72,73,74]. Secondly, the online recruiting of a survey is subject to response bias, as patients with a lower education or of an older age are less likely to participate and subjects with higher psychological distress are more likely to participate in internet research. Furthermore, our sample was characterized by a high percentage of women, probably due to a higher prevalence of IBD in European women [75] and a higher prevalence of psychological distress in women [76]. This limits the generalization of our findings. Thirdly, the sample size was relatively small, so it may not be representative of all IBD patients. Fourthly, the clinical and psychological variables (e.g., DA, disease duration) were self-reported and could not be checked against the actual medical data. It is known anecdotally that clinicians have been able to visit patients with IBD notwithstanding the pandemic restrictions and have noticed patients’ increasing states of anxiety linked to fears of not being able to be treated promptly and adequately as usual in hospitals. Patients might have reported milder or subthreshold signs of psychological distress to these physicians but not to the questionnaire. This aspect, although difficult to detect, may underestimate the actual psychological state of patients. Furthermore, although subjects with reported psychiatric disorders were excluded from the study, symptoms of anxiety and depression were not evaluated with specific assessment instruments for maximizing participation by limiting the length of the survey. Lastly, several variables may likely mediate the association between HRQoL, DA, and pandemic-related distress, such as inflammation status, diet, psychopathology, personality factors, perceived stress, social relationships, and IBD treatment. Unfortunately, because of the very nature of the study design, these factors could not be assessed, and our findings could not be controlled for, thus limiting the generalization of results. 

## 5. Conclusions

Clinical and biomedical factors such as clinical remission, treatment response, biomarker normalization, and mucosal healing have been traditionally considered the main endpoints of IBD treatment. Over the last few decades, the inclusion of the patients’ perspective has been increasingly highlighted within the conceptualization and practice of health care by using PROs, including HRQoL. In this study, the close association of HRQoL to changes in DA among IBD patients has been confirmed, over and above the weight of the COVID-19 pandemic period. During the emergency period, many concerns for clinical management of follow-up and worsened psychological status of IBD patients have been raised. However, our results suggest that, after controlling for DA, the HRQoL is more strictly related to the disease course than pandemic-related living conditions. Our findings confirm that the assessment of HRQoL is to be considered a relevant part of the health status of IBD patients jointly with DA, much more than transitory, even if dramatic, negative conditions.

## Figures and Tables

**Table 1:** DA change-related groups from T0 to T1.

	UC (*n =* 108)	CD (*n =* 113)
Improved	Patients with active disease in T0 (*n* = 60), of whom 40 were in remission in T1	Patients with active disease in T0 (*n* = 67), of whom 32 were in remission in T1
Worsened	Patients with quiescent disease in T0 (*n* = 48), of whom 19 were active in T1	Patients with quiescent disease in T0 (*n* = 46), of whom 13 were active in T1
Persistent Active disease	Patients with active disease (Mayo 3–12) both in T0 and T1 (*n* = 20)	Patients with active disease (CDAI > 150) both in T0 and T1 (*n* = 35)
Persistent Quiescent disease	Patients with quiescent disease (Mayo 0–2) both in T0 and T1 (*n* = 29)	Patients with quiescent disease (CDAI ≤ 150) both in T0 and T1 (*n* = 33)

**Table 2:** Sociodemographic and disease characteristics of the total sample and the disease activity change-related groups.

Variable	Total Sample *n =* 221	A (Improved) *n =* 72	B (Worsened) *n =* 32	C (Persistent Active) *n =* 55	D (Persistent Quiescent) *n =* 62	F/χ^2^	*p*	η^2^/Cramer’s V
Age, mean (SD)	37.88 (10.55)	39.53 (11.02)	37.72 (7.91)	38.29 (10.85)	35.76 (10.61)	1.47	0.22	0.02
Gender, *n* (%)						1.81	0.61	0.09
Male	42 (19)	16 (22.2)	5 (15.6)	12 (21.8)	9 (14.5)			
Female	179 (81)	56 (77.8)	27 (84.4)	43 (78.2)	53 (85.5)			
Educational level, *n* (%)						11.51	0.07	0.16
Secondary school	22 (10)	10 (13.9)	3 (9.4)	6 (10.9)	3 (4.8)			
High school	101 (45.7)	36 (50)	15 (46.9)	29 (52.7)	21 (33.9)			
Graduate or Post-graduate	98 (44.3)	26 (36.1)	14 (43.8)	20 (36.4)	38 (61.3)			
Disease, *n* (%)						6.25	0.10	0.17
Crohn’s disease	113 (51.1)	32 (44.4)	13 (40.6)	35 (63.6)	33 (53.2)			
Ulcerative Colitis	108 (48.9)	40 (55.6)	19 (59.4)	20 (36.4)	29 (46.8)			
Annual relapses, *n* (%)						58.72	<0.001	0.36
0–1 time	88 (39.8)	23 (31.9)	9 (28.1)	10 (18.2) *^a^*	46 (74.2)			
2–5 times	112 (50.7)	47 (65.3)	19 (59.4)	32 (58.2)	14 (22.6)			
>6 times	21 (9.5)	2 (2.8)	4 (12.5)	13 (23.6) *^b^*	2 (3.2)			
Disease duration, *n* (%)						8.02	0.24	0.13
0–5 years	62 (28.1)	22 (30.6)	10 (31.3)	13 (23.6)	17 (27.4)			
6–20 years	108 (48.9)	35 (48.6)	17 (53.1)	22 (40)	34 (54.8)			
>21 years	51 (23)	15 (20.8)	5 (15.6)	20 (36.4)	11 (17.7)			

Notes: Group A-Improved (changed disease activity from “active” in T0 to “quiescent” in T1); Group B-Worsened (changed disease activity from “quiescent” in T0 to “active” in T1); Group C-Persistent active (unchanged active disease both in T0 and T1); Group D-Persistent quiescent (unchanged quiescent disease both in T0 and T1). *^a^* Lower prevalence compared to the other DA groups (standardized deviate = −2.54). *^b^* Higher prevalence compared to the other DA groups (standardized deviate = +3.40).

**Table 3:** Clinical characteristics of the total sample and the disease activity change-related groups.

Variable, Mean (SD)	Total Sample *n =* 221	A (Improved) *n =* 72	B (Worsened) *n =* 32	C (Persistent Active) *n =* 55	D (Persistent Quiescent) *n =* 62	F	*p*	η^2^	Bonferroni Post-Hoc Test
IES-R (T0)	33.71 (16.09)	32.57 (15.21)	37.72 (17.25)	34.80 (15.54)	32.00 (16.91)	1.10	0.35	0.01	
IES-R (T1)	28.16 (15.61)	27.70 (14.65)	31.33 (18.63)	30.19 (15.98)	25.14 (14.28)	1.45	0.23	0.02	
IBDQ (T0)	130.60 (33.97)	129.51 (30.99)	125.31 (34.18)	114.47 (28.50)	149.05 (32.51)	12.37	<0.001	0.15	D > C < A = B
Bowel symptoms	42.76 (10.77)	41.93 (9.52)	40.78 (10.89)	38.20 (9.47)	48.85 (10.29)	12.23	<0.001	0.14	D > A = B = C
Systemic symptoms	17.55 (6.69)	17.67 (5.90)	16.28 (6.65)	15.18 (5.84)	20.16 (7.24)	6.38	<0.001	0.08	D > A = B = C
Emotional function	47.98 (13.04)	48.10 (12.60)	45.91 (12.06)	42.91 (11.55)	53.50 (13.19)	7.41	<0.001	0.09	D > A = B = C
Social Function	22.31 (7.40)	21.82 (6.61)	22.34 (7.55)	18.18 (6.03)	26.53 (7.09)	15.05	<0.001	0.17	D > A = B > C
IBDQ (T1)	135.33 (36.08)	139.72 (32.65)	118.10 (33.46)	112.92 (28.34)	158.50 (31.52)	23.13	<0.001	0.25	D > A = B > C
Bowel symptoms	44.42 (11.41)	46.09 (10.15)	37.70 (10.33)	37.88 (9.75)	51.57 (9.65)	23.04	<0.001	0.25	D > A = B > C
Systemic symptoms	18.20 (6.73)	19 (6.48)	15.53 (5.70)	15.15 (5.68)	21.28 (6.84)	11.01	<0.001	0.14	A > C = B < D
Emotional function	49.19 (13.54)	50.66 (12.67)	44.57 (12.83)	41.13 (10.54)	56.87 (12.62)	17.28	<0.001	0.20	D > C < A = B
Social Function	23.53 (7.79)	23.97 (6.47)	20.30 (7.67)	18.75 (6.86)	28.78 (6.62)	22.91	<0.001	0.25	D > C < A = B

Notes: IES-R = Impact of Event Scale-Revised; IBDQ = Inflammatory Bowel Disease Questionnaire. Group A-Improved (changed disease activity from “active” in T0 to “quiescent” in T1); Group B-Worsened (changed disease activity from “quiescent” in T0 to “active” in T1); Group C-Persistent active (unchanged active disease both in T0 and T1); Group D-Persistent quiescent (unchanged quiescent disease both in T0 and T1).

**Table 4:** Comparisons of HRQoL at T0 and T1 in disease activity change-related groups.

	Disease Activity-Related Groups				Time		Time * Group			Time * IES-R	
	A (Improved)	B (Worsened)	C (Persistent Active)	D (Persistent Quiescent)	F	*p*	F	*p*	η^2^	F	*p*
IBDQ, EMM (SEM)					0.90	0.34	6.02	0.001	0.08	0.27	0.60
T0	128.98 (3.23)	130.30 (4.84)	115.79 (3.66)	147.80 (3.41)							
T1	138.75 (3.35)	121.38 (5.02)	113.73 (3.80)	157.24 (3.54)							

Covariates appearing in the model are evaluated at the following values: IES-R (T0) = 33.37. Notes: estimated marginal means (EMM); standard error of the mean (SEM); IBDQ = Inflammatory Bowel Disease Questionnaire; IES-R = Impact of Event Scale-Revised.

## Data Availability

The data presented in this study are available on request from the corresponding author. The data are not publicly available due to privacy restrictions.

## References

[1] Wańkowicz P., Szylińska A., Rotter I. (2021). The impact of the COVID-19 pandemic on psychological health and insomnia among people with chronic diseases. J. Clin. Med..

[2] Cho J.H. (2008). The genetics and immunopathogenesis of inflammatory bowel disease. Nat. Rev. Immunol..

[3] Casellas F., López-Vivancos J., Vergara M., Malagelada J. (1999). Impact of inflammatory bowel disease on health-related quality of life. Dig. Dis..

[4] Mayo N. (2015). Dictionary of Quality of Life and Health Outcomes Measurement.

[5] Chan W., Shim H.H., Lim M.S., Sawadjaan F., Isaac S.P., Chuah S.W., Leong R., Kong C. (2017). Symptoms of anxiety and depression are independently associated with inflammatory bowel disease-related disability. Dig. Liver Dis..

[6] Chao C.Y., Lemieux C., Restellini S., Afif W., Bitton A., Lakatos P.L., Wild G., Bessissow T. (2019). Maladaptive coping, low self-efficacy and disease activity are associated with poorer patient-reported outcomes in inflammatory bowel disease. Saudi J. Gastroenterol..

[7] Dubinsky M.C., Dotan I., Rubin D.T., Bernauer M., Patel D., Cheung R., Modesto I., Latymer M., Keefer L. (2021). Burden of comorbid anxiety and depression in patients with inflammatory bowel disease: A systematic literature review. Expert Rev. Gastroenterol. Hepatol..

[8] Gracie D.J., Irvine A.J., Sood R., Mikocka-Walus A., Hamlin P.J., Ford A.C. (2017). Effect of psychological therapy on disease activity, psychological comorbidity, and quality of life in inflammatory bowel disease: A systematic review and meta-analysis. Lancet Gastroenterol. Hepatol..

[9] Lewis K., Marrie R.A., Bernstein C.N., Graff L.A., Patten S.B., Sareen J., Fisk J.D., Bolton J.M., CIHR team in defining the burden and managing the effects of immune-mediated inflammatory disease (2019). The prevalence and risk factors of undiagnosed depression and anxiety disorders among patients with inflammatory bowel disease. Inflamm. Bowel Dis..

[10] Knowles S.R., Graff L.A., Wilding H., Hewitt C., Keefer L., Mikocka-Walus A. (2018). Quality of life in inflammatory bowel disease: A systematic review and meta-analyses-part I. Inflamm. Bowel Dis..

[11] Knowles S.R., Keefer L., Wilding H., Hewitt C., Graff L.A., Mikocka-Walus A. (2018). Quality of life in inflammatory bowel disease: A systematic review and meta-analyses-part II. Inflamm. Bowel Dis..

[12] Bednarikova H., Kascakova N., Furstova J., Zelinkova Z., Falt P., Hasto J., Tavel P. (2021). Life stressors in patients with inflammatory bowel disease: Comparison with a population-based healthy control group in the Czech Republic. Int. J. Environ. Res..

[13] Gracie D.J., Hamlin P.J., Ford A.C. (2019). The influence of the brain-gut axis in inflammatory bowel disease and possible implications for treatment. Lancet Gastroenterol. Hepatol..

[14] Fairbrass K.M., Lovatt J., Barberio B., Yuan Y., Gracie D.J., Ford A.C. (2022). Bidirectional brain-gut axis effects influence mood and prognosis in IBD: A systematic review and meta-analysis. Gut.

[15] Van der Have M., van der Aalst K.S., Kaptein A.A., Leenders M., Siersema P.D., Oldenburg B., Fidder H.H. (2014). Determinants of health-related quality of life in Crohn’s disease: A systematic review and meta-analysis. J. Crohn’s Colitis.

[16] Zheng K., Zhang S., Wang C., Zhao W., Shen H. (2015). Health-related quality of life in Chinese patients with mild and moderately active ulcerative colitis. PloS ONE.

[17] Gibson P.R., Vaizey C., Black C.M., Nicholls R., Weston A.R., Bampton P., Sparrow M., Lawrance I.C., Selby W.S., Andrews J.M. (2014). Relationship between disease severity and quality of life and assessment of health care utilization and cost for ulcerative colitis in Australia: A cross-sectional, observational study. J. Crohn’s Colitis.

[18] Panés J., Domènech E., Aguas Peris M., Nos P., Riestra S., Juliá de Páramo B., Cea-Calvo L., Romero C., Marín-Jiménez I. (2017). Association between disease activity and quality of life in ulcerative colitis: Results from the CRONICA-UC study. J. Gastroenterol. Hepatol..

[19] Van Gennep S., De Boer N., Gielen M.E., Rietdijk S.T., Gecse K.B., Ponsioen C.Y., Duijvestein M., D’Haens G.R., Löwenberg M., De Boer A. (2021). Impaired Quality of Working Life in Inflammatory Bowel Disease Patients. J. Dig. Dis..

[20] Graff L.A., Walker J.R., Lix L., Clara I., Rawsthorne P., Rogala L., Miller N., Jakul L., McPhail C., Ediger J. (2006). The relationship of inflammatory bowel disease type and activity to psychological functioning and quality of life. Clin. Gastroenterol. Hepatol..

[21] Iglesias-Rey M., Barreiro-de Acosta M., Caamaño-Isorna F., Rodríguez I.V., Ferreiro R., Lindkvist B., González A.L., Dominguez-Munoz J.E. (2014). Psychological factors are associated with changes in the health-related quality of life in inflammatory bowel disease. Inflamm. Bowel Dis..

[22] Mussell M., Böcker U., Nagel N., Singer M.V. (2004). Predictors of disease-related concerns and other aspects of health-related quality of life in outpatients with inflammatory bowel disease. Eur. J. Gastroenterol. Hepatol..

[23] Agostini A., Ballotta D., Righi S., Moretti M., Bertani A., Scarcelli A., Sartini A., Ercolani M., Nichelli P., Campieri M. (2017). Stress and brain functional changes in patients with Crohn’s disease: A functional magnetic resonance imaging study. Neurogastroenterol. Motil..

[24] Bonaz B.L., Bernstein C.N. (2013). Brain-gut interactions in inflammatory bowel disease. Gastroenterology.

[25] Mawdsley J.E., Rampton D.S. (2005). Psychological stress in IBD: New insights into pathogenic and therapeutic implications. Gut.

[26] Becker H.M., Grigat D., Ghosh S., Kaplan G.G., Dieleman L., Wine E., Fedorak R.N., Fernandes A., Panaccione R., Barkema H.W. (2015). Living with inflammatory bowel disease: A Crohn’s and Colitis Canada survey. Can. J. Gastroenterol. Hepatol..

[27] Fonagy P., Bateman A.W. (2016). Adversity, attachment, and mentalizing. Compr. Psychiatry.

[28] Agostini A., Scaioli E., Belluzzi A., Campieri M. (2019). Attachment and Mentalizing Abilities in Patients with Inflammatory Bowel Disease. Gastroenterol. Res. Pract..

[29] Colonnello V., Agostini A. (2020). Disease course, stress, attachment, and mentalization in patients with inflammatory bowel disease. Med. Hypotheses.

[30] U.S. Department of Health and Human Services FDA Center for Drug Evaluation and Research, U.S. Department of Health and Human Services FDA Center for Biologics Evaluation and Research, U.S. Department of Health and Human Services FDA Center for Devices and Radiological Health (2006). Guidance for industry: Patient-reported outcome measures: Use in medical product development to support labeling claims: Draft guidance. Health Qual. Life Outcomes.

[31] U.S. Food and Drug Administration. https://www.fda.gov/regulatory-information/search-fda-guidance-documents/patient-reported-outcome-measures-use-medical-product-development-support-labeling-claims.

[32] Kappelman M.D., Long M.D., Martin C., DeWalt D.A., Kinneer P.M., Chen W., Lewis J.D., Sandler R.S. (2014). Evaluation of the patient-reported outcomes measurement information system in a large cohort of patients with inflammatory bowel diseases. Clin. Gastroenterol. Hepatol..

[33] Janowitz T., Gablenz E., Pattinson D., Wang T.C., Conigliaro J., Tracey K., Tuveson D. (2020). Famotidine use and quantitative symptom tracking for COVID-19 in non-hospitalised patients: A case series. Gut.

[34] Pinto S., Loddo E., Paba S., Favale A., Chicco F., Onali S., Usai P., Fantini M.C. (2021). Crohn’s disease and ulcerative colitis patient-reported outcomes signs and symptoms for the remote management of inflammatory bowel disease during the COVID-19 pandemic. J. Patient-Rep..

[35] Al-Ani A.H., Prentice R.E., Rentsch C.A., Johnson D., Ardalan Z., Heerasing N., Garg M., Campbell S., Sasadeusz J., Macrae F.A. (2020). Review article: Prevention, diagnosis and management of COVID-19 in the IBD patient. Aliment. Pharmacol. Ther..

[36] Graff L.A., Fowler S., Jones J.L., Benchimol E.I., Bitton A., Huang J.G., Kuenzig M.E., Kaplan G.G., Lee K., Mukhtar M.S. (2021). Crohn’s and Colitis Canada’s 2021 Impact of COVID-19 and Inflammatory Bowel Disease in Canada: Mental Health and Quality of Life. J. Can. Assoc. Gastroenterol..

[37] Harris R.J., Downey L., Smith T.R., Cummings J.F., Felwick R., Gwiggner M. (2020). Life in lockdown: Experiences of patients with IBD during COVID-19. BMJ Open Gastroenterol..

[38] Kim K.O., Jang B.I. (2022). Management of inflammatory bowel disease in the COVID-19 era. Intest. Res..

[39] De Bock E., Filipe M.D., Meij V., Oldenburg B., Van Schaik F., Bastian O.W., Fidder H.F., Vriens M.R., Richir M.C. (2021). Quality of life in patients with IBD during the COVID-19 pandemic in the Netherlands. BMJ Open Gastroenterol..

[40] Eşkazan T., Bakkaloğlu O.K., Durcan E., Kurt E.A., Önal U., Candan S., Tuncer M., Demirel Ö., Hatemi İ., Erzin Y. (2022). The psychological effects of COVID-19 pandemic in patients with inflammatory bowel disease. Turk J. Gastroenterol..

[41] Gavrilescu O., Prelipcean C.C., Dranga M., Popa I.V., Mihai C. (2022). Impact of COVID-19 pandemic on the quality of life of IBD patients. Medicina.

[42] Stone M.L., Feng M., Forster E.M. (2022). COVID-19 pandemic increased anxiety among patients with inflammatory bowel disease: A patient survey in a tertiary referral center. Dig. Dis. Sci..

[43] Trindade I.A., Ferreira N.B. (2021). COVID-19 pandemic’s effects on disease and psychological outcomes of people with inflammatory bowel disease in portugal: A preliminary research. Inflamm. Bowel Dis..

[44] Azzam N.A., Aljebreen A., Almuhareb A., Almadi M.A. (2020). Disability and quality of life before and during the COVID-19 outbreak: A cross-sectional study in inflammatory bowel disease patients. Saudi J. Gastroenterol..

[45] Wang H., Tu L., Li Y., Bai T., Zou K., Xiao F., Li J., Chen M., Zhang H., Li G. (2020). The symptoms and medications of patients with inflammatory bowel disease in Hubei province after COVID-19 epidemic. J. Immunol. Res..

[46] Paulides E., Pasma A., Erler N.S., van Eijk R., de Vries A.C., van der Woude C.J. (2022). Impact of the coronavirus disease pandemic on health-related quality of life of patients with inflammatory bowel disease. Dig. Dis. Sci..

[47] Conti C., Rosa I., Zito L., Grossi L., Efthymakis K., Neri M., Porcelli P. (2021). Influence of the COVID-19 outbreak on disease activity and quality of life in inflammatory bowel disease patients. Front. Psychiatry.

[48] World Medical Association (2013). World medical association declaration of helsinki: Ethical principles for medical research involving human subjects. JAMA.

[49] Schroeder K.W., Tremaine W.J., Ilstrup D.M. (1987). Coated oral 5-aminosalicylic acid therapy for mildly to moderately active ulcerative colitis. A randomized study. N. Engl. J. Med..

[50] Dhanda A.D., Creed T.J., Greenwood R., Sands B.E., Probert C.S. (2012). Can endoscopy be avoided in the assessment of ulcerative colitis in clinical trials?. Inflamm. Bowel Dis..

[51] Best W.R., Becktel J.M., Singleton J.W., Kern F. (1976). Development of a Crohn’s disease activity index. National cooperative crohn’s disease study. Gastroenterology.

[52] Best W.R. (2006). Predicting the Crohn’s disease activity index from the Harvey-Bradshaw Index. Inflamm. Bowel Dis..

[53] Craparo G., Faraci P., Rotondo G., Gori A. (2013). The impact of event scale-revised: Psychometric properties of the Italian version in a sample of flood victims. Neuropsychiatr. Dis. Treat..

[54] Weiss D.S., Marmar C.R., Wilson J.P., Keane T.M. (1997). The impact of event scale-revised. Assessing Psychological Trauma and PTSD.

[55] Creamer M., Bell R., Failla S. (2003). Psychometric properties of the impact of event scale-revised. Behav. Res. Ther..

[56] Forte G., Favieri F., Tambelli R., Casagrande M. (2020). COVID-19 pandemic in the italian population: Validation of a post-traumatic stress disorder questionnaire and prevalence of PTSD symptomatology. Int. J. Environ. Res. Public Health.

[57] Guyatt G., Mitchell A., Irvine E.J., Singer J., Williams N., Goodacre R., Tompkins C. (1989). A new measure of health status for clinical trials in inflammatory bowel disease. Gastroenterology.

[58] Cohen J. (1973). Eta-squared and partial eta-squared in fixed factor anova designs. Educ. Psychol. Meas..

[59] Yu M., Ye Z., Chen Y., Qin T., Kou J., Tian D., Xiao F. (2020). Questionnaire assessment helps the self-management of patients with inflammatory bowel disease during the outbreak of Coronavirus Disease 2019. Aging.

[60] Casellas F., López-Vivancos J., Casado A., Malagelada J.R. (2002). Factors affecting health related quality of life of patients with inflammatory bowel disease. Qual. Life Res..

[61] Dorrian A., Dempster M., Adair P. (2009). Adjustment to inflammatory bowel disease: The relative influence of illness perceptions and coping. Inflamm. Bowel Dis..

[62] Guan T., Santacroce S.J., Chen D.G., Song L. (2020). Illness uncertainty, coping, and quality of life among patients with prostate cancer. Psycho-oncology.

[63] Niv G., Bar Josef S., Ben Bassat O., Avni I., Lictenstein L., Niv Y., Barnoy S. (2017). Quality of life and uncertainty in Crohn’s disease. Qual. Life Res..

[64] Mason J.W. (1968). A review of psychoendocrine research on the pituitary-adrenal cortical system. Psychosom. Med..

[65] Peters A., McEwen B.S., Friston K. (2017). Uncertainty and stress: Why it causes diseases and how it is mastered by the brain. Prog. Neurobiol..

[66] Gamwell K.L., Baudino M.N., Bakula D.M., Sharkey C.M., Roberts C.M., Grunow J.E., Jacobs N.J., Gillaspy S.R., Mullins L.L., Chaney J.M. (2018). Perceived illness stigma, thwarted belongingness, and depressive symptoms in youth with inflammatory bowel disease (IBD). Inflamm. Bowel Dis..

[67] Lasker J.N., Sogolow E.D., Olenik J.M., Sass D.A., Weinrieb R.M. (2010). Uncertainty and liver transplantation: Women with primary biliary cirrhosis before and after transplant. Women Health.

[68] Parker P.A., Alba F., Fellman B., Urbauer D.L., Li Y., Karam J.A., Tannir N., Jonasch E., Wood C.G., Matin S.F. (2013). Illness uncertainty and quality of life of patients with small renal tumors undergoing watchful waiting: A 2-year prospective study. Eur. Urol..

[69] Madar H., Bar-Tal Y. (2009). The experience of uncertainty among patients having peritoneal dialysis. J. Adv. Nurs..

[70] Lin L., Chiang H.H., Acquaye A.A., Vera-Bolanos E., Gilbert M.R., Armstrong T.S. (2013). Uncertainty, mood states, and symptom distress in patients with primary brain tumors: Analysis of a conceptual model using structural equation modeling. Cancer.

[71] Nanton V., Munday D., Dale J., Mason B., Kendall M., Murray S. (2016). The threatened self: Considerations of time, place, and uncertainty in advanced illness. Br. J. Health Psychol..

[72] Buselli R., Corsi M., Baldanzi S., Chiumiento M., Del Lupo E., Dell’Oste V., Bertelloni C.A., Massimetti G., Dell’Osso L., Cristaudo A. (2020). Professional quality of life and mental health outcomes among health care workers exposed to Sars-Cov-2 (COVID-19). Int. J. Environ. Res. Public Health.

[73] Jeppesen S.S., Bentsen K.K., Jørgensen T.L., Holm H.S., Holst-Christensen L., Tarpgaard L.S., Dahlrot R.H., Eckhoff L. (2021). Quality of life in patients with cancer during the COVID-19 pandemic—A Danish cross-sectional study (COPICADS). Acta Oncol..

[74] Liu C.H., Stevens C., Conrad R.C., Hahm H.C. (2020). Evidence for elevated psychiatric distress, poor sleep, and quality of life concerns during the COVID-19 pandemic among U.S. young adults with suspected and reported psychiatric diagnoses. Psychiatry Res..

[75] Greuter T., Manser C., Pittet V., Vavricka S.R., Biedermann L., on Behalf of Swiss IBDnet, an official working group of the Swiss society of gastroenterology (2020). Gender differences in inflammatory bowel disease. Digestion.

[76] Holingue C., Budavari A.C., Rodriguez K.M., Zisman C.R., Windheim G., Fallin M.D. (2020). Sex differences in the gut-brain axis: Implications for mental health. Curr. Psychiatry Rep..

